# Development of siRNA-Loaded Lipid Nanoparticles Targeting Long Non-Coding RNA LINC01257 as a Novel and Safe Therapeutic Approach for t(8;21) Pediatric Acute Myeloid Leukemia

**DOI:** 10.3390/pharmaceutics13101681

**Published:** 2021-10-14

**Authors:** Patrick Connerty, Ernest Moles, Charles E. de Bock, Nisitha Jayatilleke, Jenny L. Smith, Soheil Meshinchi, Chelsea Mayoh, Maria Kavallaris, Richard B. Lock

**Affiliations:** 1 Children’s Cancer Institute, Lowy Cancer Research Centre, UNSW Sydney, Sydney, NSW 2052, Australia; pconnerty@ccia.org.au (P.C.); EMoles@ccia.org.au (E.M.); CDeBock@ccia.org.au (C.E.d.B.); njayatilleke@ccia.org.au (N.J.); CMayoh@ccia.org.au (C.M.); MKavallaris@ccia.org.au (M.K.); 2 School of Women’s and Children’s Health, UNSW Sydney, Sydney, NSW 2052, Australia; 3 University of New South Wales Centre for Childhood Cancer Research, UNSW Sydney, Sydney, NSW 2052, Australia; 4 Australian Centre for Nanomedicine, ARC Centre of Excellence in Bio-Nano Science and Technology, UNSW Sydney, Sydney, NSW 2052, Australia; 5 Clinical Research Division, Fred Hutchinson Cancer Research Center, Seattle, WA 98109, USA; jlsmith3@fredhutch.org (J.L.S.); smeshinc@fredhutch.org (S.M.); 6 Division of Pediatric Hematology/Oncology, University of Washington, Seattle, WA 98109, USA

**Keywords:** lipid-based nanoparticles, long non-coding RNA, RNA interference, nanomedicine, nanoparticle-assisted siRNA delivery

## Abstract

Standard of care therapies for children with acute myeloid leukemia (AML) cause potent off-target toxicity to healthy cells, highlighting the need to develop new therapeutic approaches that are safe and specific for leukemia cells. Long non-coding RNAs (lncRNAs) are an emerging and highly attractive therapeutic target in the treatment of cancer due to their oncogenic functions and selective expression in cancer cells. However, lncRNAs have historically been considered ‘undruggable’ targets because they do not encode for a protein product. Here, we describe the development of a new siRNA-loaded lipid nanoparticle for the therapeutic silencing of the novel oncogenic lncRNA LINC01257. Transcriptomic analysis of children with AML identified LINC01257 as specifically expressed in t(8;21) AML and absent in healthy patients. Using NxGen microfluidic technology, we efficiently and reproducibly packaged anti-LINC01257 siRNA (LNP-si-LINC01257) into lipid nanoparticles based on the FDA-approved Patisiran (Onpattro^®^) formulation. LNP-si-LINC01257 size and ζ-potential were determined by dynamic light scattering using a Malvern Zetasizer Ultra. LNP-si-LINC01257 internalization and siRNA delivery were verified by fluorescence microscopy and flow cytometry analysis. lncRNA knockdown was determined by RT-qPCR and cell viability was characterized by flow cytometry-based apoptosis assay. LNP-siRNA production yielded a mean LNP size of ~65 nm with PDI ≤ 0.22 along with a >85% siRNA encapsulation rate. LNP-siRNAs were efficiently taken up by Kasumi-1 cells (>95% of cells) and LNP-si-LINC01257 treatment was able to successfully ablate LINC01257 expression which was accompanied by a significant 55% reduction in total cell count following 48 h of treatment. In contrast, healthy peripheral blood mononuclear cells (PBMCs), which do not express LINC01257, were unaffected by LNP-si-LINC01257 treatment despite comparable levels of LNP-siRNA uptake. This is the first report demonstrating the use of LNP-assisted RNA interference modalities for the silencing of cancer-driving lncRNAs as a therapeutically viable and non-toxic approach in the management of AML.

## 1. Introduction

Acute myeloid leukemia (AML) is a molecularly heterogeneous disease that is defined by the uncontrolled rapid expansion of immature myeloid blast cells. AML is the deadliest acute leukemia in children, with an overall survival rate of only 70%, which has not substantially improved for decades [1]. Multiple rounds of chemotherapy remain the gold standard treatment option for AML, although current therapies lack specificity against leukaemic cells and severely affect healthy tissues [2]. CAR-T cell therapy and small molecule inhibitors (SMIs) have emerged as efficacious approaches to target AML cells and specific proteins that drive the disease [3]. However, these therapies suffer from off-target toxicity and their use is limited to only a small fraction of AML patients [3,4]. Consequently, to improve treatment outcomes in children with AML, it is necessary to explore new treatments that are specific to leukemia cells and thereby safe for healthy cells. 

Chromosomal translocations form fusion proteins that can induce gene expression either directly, or via new enhancers [5]. These translocation events can dysregulate the expression of genes involved in self-renewal, survival and chemoresistance. Consequently, fusion proteins or altered pro-survival signaling pathways have been identified as triggers of several cancers, including AML [5], and represent cancer-specific and highly attractive therapeutic targets. Pioneering studies have demonstrated that targeting fusion proteins using SMIs can reduce cancer progression [6]. Nonetheless, chromosomal aberrations commonly involve multiple fusion partners and these can occur at multiple breakpoints in the genome, resulting in the expression of a plethora of fusion proteins that can limit the clinical utility of SMIs [7]. Despite such structural heterogeneity, different fusion proteins can dysregulate the expression of common signaling pathways in the cancer cell, thereby reducing the number of potential targets to a few key genes [8]. One group of such key genes are non-coding genes, a heterogenous group of genes that lack any protein-coding potential [9]. 

Originally thought of as ‘junk’ DNA, non-coding genes along with their noncoding RNA products are known to have well-established functions in the regulation of gene expression and chromatin-based processes [10]. In particular, >200 nucleotide long non-coding RNAs (lncRNAs) play a crucial role in cancer cell cycle regulation and survival, either acting directly as oncogenes or indirectly via interaction with other oncogenes [10]. However, the majority of lncRNAs still have unknown biological functions and further research is needed to characterize their roles in both healthy cells and diseases. 

Recent studies have identified several lncRNAs as drivers of AML in mice and have demonstrated that their silencing through RNA interference therapy (RNAi) can inhibit AML progression [11]. In this study, we identified LINC01257, a type of lncRNA with no previously reported function, in children with AML that specifically harbor the t(8;21) translocation (a subtype of leukemia also known as AML1-ETO). Indicative of an oncogenic role, we demonstrate that higher expression of LINC01257 is associated with poorer survival and increased leukemia proliferation. Its oncogenic profile along with its specific expression on t(8;21) AML cells represent a unique opportunity to silence LNC01257 expression as a new approach to specifically target pediatric AML. 

Lipid-based nanoparticles (LNPs) represent the most successful vector for safe and effective intracellular delivery of RNA interference drugs, such as small interfering RNA (siRNA). Of note, LNPs have allowed RNA interference therapy to achieve clinical success, with the first siRNA-loaded LNP therapeutic (Patisiran) being granted FDA approval in 2018 for the management of hereditary amyloidosis [12,13]. LNP-assisted siRNA delivery has also been shown to be effective at silencing expression of oncogenes in AML and other cancers [14,15]. However, no work has yet explored this technology to target lncRNAs in AML

Microfluidic-based systems are commonly used to efficiently generate siRNA-LNP with high reproducibility and scalability [16,17]. Recently, NxGen microfluidics systems have emerged to further scale-up production without losing silencing efficacy [18]. Here, using NxGen microfluidic technology, we generated siRNA-LNPs to silence LINC01257 expression in t(8;21) Kasumi-1 AML cells. Anti-LINC01257 siRNA-loaded LNPs showed rapid accumulation in Kasumi-1 AML cells, inducing apoptosis and inhibiting proliferation while causing no significant effects on the viability of healthy peripheral blood mononuclear cells (PBMCs). This work identifies the lncRNA LINC01257 as a novel therapeutic target in pediatric t(8;21) AML and provides proof-of-concept for the silencing of AML-specific lncRNAs as a new treatment modality for future investigation in the management of pediatric AML.

## 2. Materials and Methods

### 2.1. Materials

Cholesterol and the lipids 1,2-distearoyl-sn-glycero-3-phosphocholine (DSPC), 1,2-Dimyristoyl-rac-glycero-3-methoxypolyethylene glycol-2000 (DMG-PEG), and 1,2-dioleoyl-sn-glycero-3-phosphoethanolamine-N-[lissamine rhodamine B sulfonyl] (DOPE-Rho) (all ≥99% purity according to thin-layer chromatography analysis) were purchased from Merck & Co., Inc (Kenilworth, N.J., USA). D-Lin-MC3-DMA (≥98% purity according to thin-layer chromatography analysis) was purchased from Assay Matrix Pty Ltd. (Ivanhoe North, VIC, Australia). Lincode siRNA targeting LINC01257 (Cat # 116437) was purchased from Horizon Discovery (Cambridge, UK). Non-targeting SCR siRNA, siRNA targeting ADARB2-AS1 and FAM-labelled siRNA were purchased from GenePharma (Shanghai, China).

### 2.2. Differential Expression and Survival Analysis

RNA-seq data from the Therapeutically Applicable Research to Generate Effective Treatments (TARGET) (https://ocg.cancer.gov/programs/target, accessed on 3 November 2019) initiative, phs000465, were downloaded. Raw counts and TPM values were obtained for 285 pediatric AML patients. Differential gene expression analysis was performed using the R package edgeR [19] with a fold change ≥|1.5| and FDR of <0.5 used to identify significant genes between patients carrying the t(8;21) translocation and those with wild type (WT) karyotype presenting no fusion. Survival data were provided as part of the AAML1031 study which catalogues full RNA-seq and clinical data for 1130 pediatric AML patients. Survival analysis was performed using the R package ‘survival’ [20]. Patients carrying the t(8;21) translocation were separated into high and low expression as determined by TPM value for each gene of interest where high expression referred to the top 75% of patients and low expression the bottom 25% of patients (Q1 split). Cox’s proportional hazards regression models were fitted to the survival data. A custom R script was used to perform a log2 TPM comparison between groups separated by cytogenetic subtype. Student’s t-test was performed for statistical comparison.

### 2.3. ChIP-seq Data

ChIP-seq data were downloaded from GEO and analyzed using Integrative Genomics Viewer (IGV). H3K9K14 and H3K27me3 data were downloaded from dataset GSE62847. H4ac data were downloaded from dataset GSM2026058. H3K4me2 data were downloaded from dataset GSM1844449. H3K4me3 data were downloaded from the GSM1534445 and GSM2166074 datasets and the ENCODE 2012 dataset.

### 2.4. Cell Culture

Human leukemia cell lines were cultured in RPMI-1640 medium supplied by Thermo Fisher Scientific (Waltham, MA, USA) containing 20% fetal bovine serum (FBS). Cells were cultured at 37 °C and 5% CO_2_. Cells in the logarithmic growth phase were prepared for subsequent experiments as described. Human peripheral blood mononuclear cells (PBMCs) were supplied cryopreserved by Lonza Australia (Mount Waverley, VIC, Australia). PBMCs were isolated from peripheral blood of healthy donors by apheresis machine, depleted of red blood cells and washed to remove platelets prior to cryopreservation. On arrival, PBMCs were thawed following supplier recommendations and cultured in lymphocyte growth medium-3 (3 × 10^6^ cells/mL) for a maximum of 72 h at 37 °C and 5% CO_2_. In each in vitro assay using PBMCs, these were immunophenotyped to identify B cells, T cells and monocytes by 30 min staining on ice with a mixture of antibodies containing BV421 anti-human CD19, V500 anti-human-CD3 and Alexa Fluor 700 anti-human CD14, 1 µg/mL of each.

### 2.5. AML Patient Samples

Cryopreserved mononuclear cells (MNCs) from 7 pediatric patients diagnosed with AML were provided by the Children’s Cancer Institute Tumour Bank. Healthy pediatric RNA obtained from CD34+ cells was kindly provided by the laboratory of Prof. John Pimanda (Lowy Cancer Research Centre and the Prince of Wales Clinical School, UNSW, Sydney, Australia). Patient samples were acquired under human ethics (LNR/14/SCHN/392) approval from Sydney Children’s Hospital Network Human Research Ethics Committee (SCHN HREC).

### 2.6. Cell Fractionation

Kasumi-1 cells were fractionated using the PARIS™ Kit (Thermo Fisher Scientific) as per the manufacturer’s instructions. RP18S and MALAT1 were used as cytoplasmic and nuclear controls, respectively, for RT-qPCR analysis. 

### 2.7. RNA Isolation and qPCR

Total RNA was extracted by Qiagen RNeasy mini kit (Qiagen, Hilden, Germany) and AllPrep DNA/RNA/miRNA universal kit (Qiagen) for cell lines and patient samples, respectively, as per the manufacturer’s instructions. cDNA was synthesized using Superscript III reverse transcriptase kit (Thermo Fisher Scientific). qPCRs were run on a Bio-Rad CFX96 Touch Real-Time PCR (Bio-Rad, Hercules, CA, USA). TBP was used as a housekeeping gene. The 2^−ΔΔCt^ method was used to analyze the relative gene expression. Primer sequences are as follows:

LINC01257-F GGCAAAGGATTCGAAGAGAC 

LINC01257-R TCAGCATGAGTGGAAAGTG

 

ADARB2-AS1-F CCCATGTCAACAGAGTTGTGTG

ADARB2-AS1-R GAGGGGTTGTGCAGAAAATC

 

TBP-F CGGCTGTTTAACTTCGCTTC 

TBP-R CACACGCCAAGAAACAGTGA

 

RPS18-F TAGCCTTTGCCATCACTGCC

RPS18-R CATGAGCATATCTTCGGCCC

 

MALAT1-F GAATTGCGTCATTTAAAGCCTAGTT

MALAT1-R GTTTCATCCTACCACTCCCAATTAAT

### 2.8. siRNA Electroporation, Cell Counts and Apoptosis Assay

Next, 2 × 10^6^ Kasumi-1 cells were electroporated in 400 µL of RPMI-1640 (Thermo Fisher Scientific) containing 400 nM non-targeting SCR siRNA, si-ADARB2-AS1 or si-LINC01257 at 300 V for 10 ms using a Bio-Rad Genepulser X (Bio-Rad). Following electroporation, cells were cultured in 2 mL of RPMI-1640 containing 20% FBS at 37 °C and 5% CO_2_ for 1 h. Cells were then counted and seeded at the indicated density for subsequent experiments. For cell count analysis, Kasumi-1 cells were seeded in a 12 well plate at 1 × 10^5^ cells/mL in RPMI-1640 containing 5% FBS. Cells were incubated at 37 °C and 5% CO_2_ and viable cell number were counted using the Trypan blue exclusion method at the indicated time points. For apoptosis assays, Kasumi-1 cells were collected and resuspended in 300 µL 1X Annexin Binding buffer and stained with 1X binding buffer containing 1:20 pro-apoptotic marker Annexin V APC (BD Biosciences, Macquarie Park, NSW, Australia) and 1:20 17AAD (BD Biosciences) for 20 min under dark conditions. Cell death was determined using flow cytometry analysis on a FACS Canto II (BD Biosciences). Cells that were both Annexin V^−^ and 7AAD^−^ were considered viable for further analysis.

### 2.9. Generation of LNP-siRNA

LNP-si-SCR and LNP-si-LINC were prepared through NxGen microfluidics using a NanoAssemblr™ Spark^®^ instrument (Precision Nanosystems, Vancouver, BC, Canada). Briefly, one volume of a mixture of lipids in pure ethanol (D-Lin-MC3, DSPC, cholesterol, DMG-PEG, DOPE-Rho; 14.8 mM total lipid) and three volumes of siRNA in 25 mM acetate buffer at pH 4.0 (12:1 wt/wt lipid to siRNA ratio) were loaded separately into a Spark^®^ microfluidic cartridge and mixed at a controlled flow rate under sterile conditions (3 mL/min and 9 mL/min for organic and aqueous streams, respectively). The resulting LNP-siRNA suspension was then diluted 1:4 in 0.1 M phosphate buffer, pH 7.4. Prior to addition to cell cultures, LNP-siRNA suspensions were further diluted in PBS (pH 7.4) or culture media to lessen traces of ethanol to non-toxic levels (<0.5% *v*/*v*). LNP-siRNA concentrations in this article always refer to LNP-entrapped siRNA (µM). LNP-siRNA size and ζ-potential were determined by dynamic light scattering using a Malvern Zetasizer Ultra (Malvern Ltd., Malvern, United Kingdom). siRNA encapsulation (%) in LNPs was determined by Quant-iT™ RiboGreen™ RNA Assay (Thermo Fisher Scientific) in the presence and absence of Triton X-100 (0.5% *v*/*v*) as reported in published methods [21].

### 2.10. Cryogenic Electron Microscopy Imaging 

A volume of 4.5 mL of each LNP sample was applied to glow-discharged copper grids (Quantifoil R1.2/1.3, Quantifoil Micro Tools), blotted for 3 s at 4 °C with 90% humidity and plunged into liquid ethane using a Lecia EM GP device (Leica Microsystem, Wetzlar, Germany). The vitrified samples were stored in liquid nitrogen (−196 °C) prior to cryo-electron microscopy (EM) analysis. Cryo-EM data were collected on a Thermo Fisher Talos Arctica transmission electron microscope operated at 200 kV acceleration voltage. Images were recorded on a Falcon III detector (Thermo Fisher Scientific).

### 2.11. Fluorescence Microscopy Analysis of LNP-siRNA Uptake in AML Cells

Live Kasumi-1 cells (6 × 10^5^ cells/mL) were washed twice with RPMI-1640 containing 20% FBS and incubated with LNPs encapsulating FAM-labelled SCR siRNA (250 nM siRNA) (Genepharma) for 3 h at 37 °C and 5% CO_2_. Cells were washed with PBS and cell nuclei and plasma membrane were stained with 2 µg/mL Hoechst 33342 and 5 µg/mL CellMask™ Deep Red Plasma Membrane Stain (Thermo Fisher Scientific) for 15 min in PBS. Excess dye was removed by washing twice with PBS, and 5 × 10^5^ cells in PBS were finally deposited onto Nunc™ Lab-Tek™ 8-well Chambered Cover glass slides (Thermo Fisher Scientific) for 30 min. Confocal microscopic imaging of live cell-internalized LNP-siRNA was carried out in a Leica TCS SP8 DLS inverted laser scanning confocal microscope equipped with a DMI6000 motorized stage, lasers UV DMOD (405 nm), Argon (458/476/488/496/514 nm), DPSS (561 nm), HeNe (633 nm) and a 63× oil (NA 1.4) immersion objective. Hoechst (nuclei), Rhodamine (LNP vector), FITC (siRNA cargo) and CellMask Deep Red (plasma membrane) fluorescence were acquired sequentially using 405 nm, 488 nm, 561 nm and 633 nm laser lines, and emission detection ranges 425–480 nm, 500–600 nm, 605–630 nm and 640–670 nm, respectively, with the confocal pinhole set at 1 Airy unit. Images were acquired at 100 Hz in a 1024 × 1024 pixels format.

### 2.12. LNP-siRNA Association with Cells

To determine LNP-siRNA association with AML cells and PBMCs, cells were seeded at 6 × 10^5^ cells/mL and 1.5 × 10^6^ cells/mL, respectively, and incubated for 3 h at 37 °C (5% CO_2_) with LNPs encapsulating FITC-labelled SCR siRNA at the indicated siRNA concentrations. Excess LNP-siRNA was removed by washing with PBS. PBMCs were finally incubated with 1:50 BV421 mouse anti-human CD19 and 1:50 APC-H7 mouse anti-human CD3 in PBS for 30 min to label the B-cell and T-cell lymphocyte populations, respectively. The % of cells positive for LNP-siRNA uptake were finally assessed by flow cytometry using a BD LSRFortessa™ SORP cell analyzer (BD Biosciences) and Flowjo software for data analysis.

### 2.13. LNP-siRNA Treatment of AML Cells and PBMCs

Next, 2.5 × 10^5^ Kasumi-1 cells were incubated with either LNP-si-SCR or LNP-si-LINC01257 and cultured in 500 µL of RPMI-1640 containing 20% FBS at 37 °C and 5% CO_2_ at concentrations and time points indicated. Meanwhile, 3 × 10^6^ cells/mL PBMCs were treated with LNP-si-SCR or LNP-si-LINC in 1 mL of LGM-3 media at 37 °C and 5% CO_2_ at concentrations at the time points indicated. For cell counts, equal volumes of Kasumi-1 cells were spiked with BD Liquid counting beads (BD Biosciences) and flow cytometry was used to determine cells/mL as outlined in the manufacturer’s instructions. For viability staining, Kasumi-1 cells were collected and resuspended in 300 µL 1× Annexin Binding buffer and stained with 1× Binding Buffer (BB) (BD Biosciences) containing 1:20 pro-apoptotic marker Annexin V APC (BD Biosciences) and either 1:20 7AAD (BD Biosciences) or 1:100 Cell Viability Dye SYTOX™ Green Nuclei Acid Stain for 20 min under dark conditions. Cell death was determined using flow cytometry analysis on a FACS Canto II (BD Biosciences). Cells that were both Annexin V^−^ and 17AAD^−^ or Sytox^−^ were considered viable for further analysis. PBMCs were suspended to a concentration of 3 × 10^6^ cells/mL and washed in 1 mL of 1× BB, stained with 200 µL 1× BB containing 1:50 BV421 mouse anti-human CD19 (B-cells labelling), 1:50 V500 mouse anti-human CD3 (T-cells labelling) and 5 µg/mL SYTOX™ Green Nuclei Acid Stain and 1:20 Annexin-APC for 20 min. Excess dye was removed by washing with 1 mL BB and cell death was determined by flow cytometry (BD LSRFortessa™ SORP) and Flowjo software for data analysis.

### 2.14. Statistical Analysis

Unless stated otherwise, data are presented as the mean ± standard error of 3 biological replicates. Statistical analysis was performed using GraphPad Prism 8 software or R. Differences were considered significant at *p* < 0.05.

## 3. Results

### 3.1. Identification of lncRNAs Specific to Pediatric t(8;21) AML

**Figure 1:**
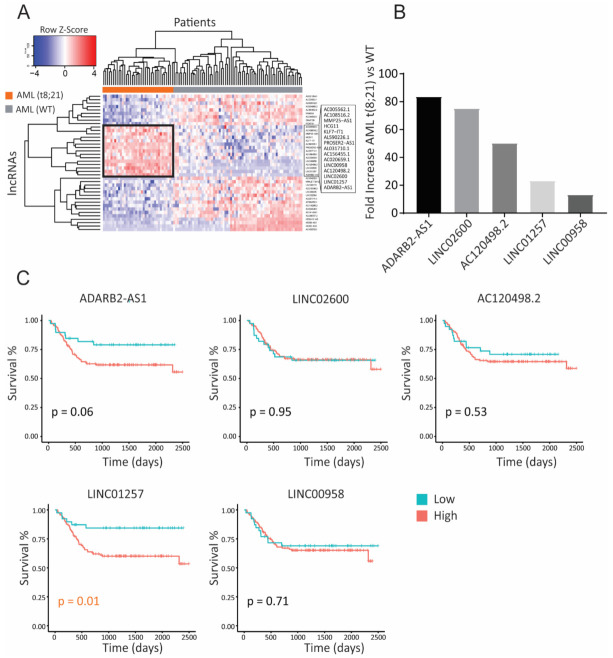
lncRNA expression is specific to t(8;21) AML and impacts patient survival. (**A**) Heat map of differential lncRNA expression analysis from TARGET data revealing a subset of lncRNAs overexpressed in AML t(8;21) patients (black rectangle). (**B**) Fold increase in expression of the top 5 highly expressed lncRNAs from Figure 1A compared to normal karyotype (WT) patients. (**C**) Kaplan–Meier curve analysis of the top 5 lncRNAs in t(8;21) patients from the AML1031 dataset. High vs low patients split by Q1 split.

Previous studies have demonstrated the relationship between the inherent role of chromosomal translocations in inducing the expression of genes in a cancer-specific manner [5,9]. In this work, we explored whether the presence of chromosomal translocations in children with AML can lead to over-expression of oncogenic lncRNAs and, if so, which lncRNAs correlate with poorer outcomes. As a proof of concept, we studied children with the t(8;21) AML translocation [22], one of the most common translocations in pediatric AML, and looked for lncRNAs that are differentially expressed in these patients when compared to children with AML lacking chromosomal abnormalities, referred here to as wild type (WT) AML. Utilizing the Therapeutically Applicable Research to Generate Effective Treatments (TARGET) study (phs000465), we identified 15 lncRNAs that are differentially expressed in children with t(8;21)-positive compared to those with WT AML (Figure 1A). Out of these, ADARB2-AS1, LINC02600, AC120498.2, LINC01257 and LINC00958 had >10-fold increased expression in t(8;21) AML patients (Figure 1B) and interestingly, all have no known biological function in AML, with only LINC00958 having oncogenic roles reported in other solid malignancies [23,24,25]. Together, this highlights the major limitations we have in our knowledge regarding the role of lncRNAs in AML. To determine whether the expression of these five lncRNAs is associated with AML patient outcome, we analyzed the AAML1031 dataset, which contains clinical outcome data for over 1000 uniformly treated pediatric AML patients. Kaplan–Meier curve analysis revealed that expression of lncRNAs LINC01257 and ADARB2-AS1 correlated with poorer event-free survival, with higher expression levels resulting in <60% event-free survival over time (Figure 1C). However, of these two, only LINC01257 expression led to significant differences in survival (*p* = 0.06 and *p* = 0.01 for ADARB2-AS1 and LINC01257, respectively). 

### 3.2. LINC01257 Knockdown Impairs AML Proliferation and Reduces Cell Viability In Vitro

**Figure 2:**
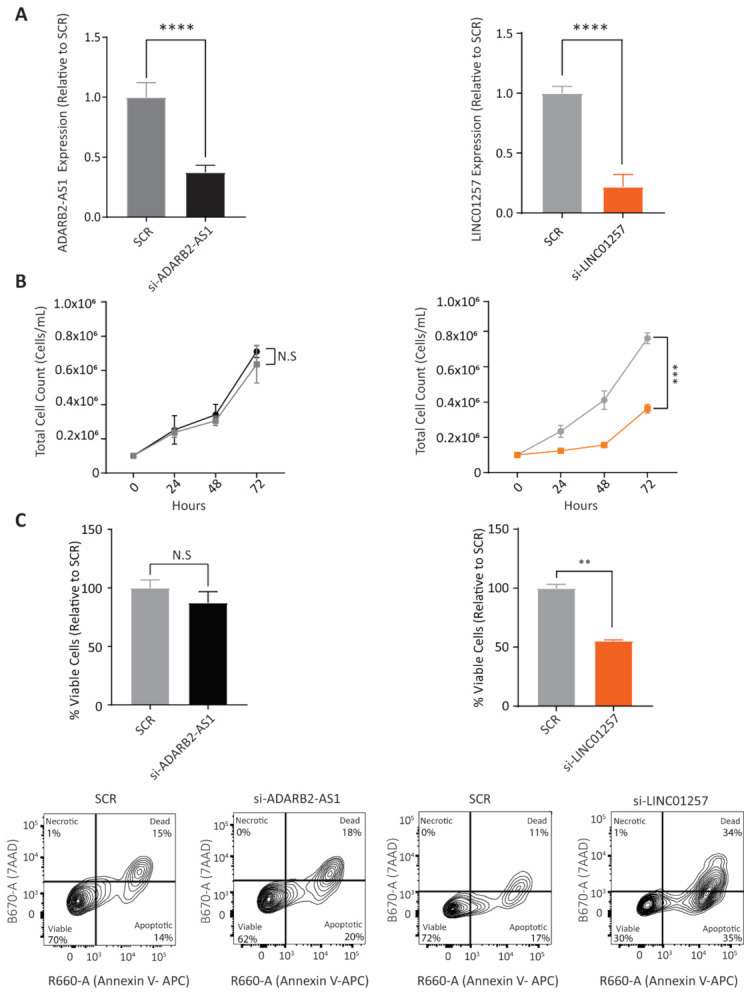
LINC01257 expression regulates Kasumi-1 cell proliferation and viability. (**A**) RT-qPCR of ADARB2-AS1 and LINC01257 expression following electroporation of si-SCR, si-ADARB2-AS1 or si-LINC01257. **** = *p* < 0.0001. *n* = 3. (**B**) Cell proliferation of Kasumi-1 transfected with si-SCR (grey), si-ADARB2-AS1 (black) or si-LINC01257 (orange) over 72 h. N.S = no significance. *** = *p* < 0.001. *n* = 3. (**C**) Percentage of viable Kasumi-1 cells 48 h following electroporation with si-SCR, si-ADARB2-AS1 and si-LINC01257. N.S = no significance. ** = *p* < 0.01. *n* = 3. Representative flow cytometry plots of Kasumi-1 cells stained with Annexin V/7AAD are shown in bottom panels.

We next sought to determine whether changes in LINC01257 or ADARB2-AS1 lncRNA expression could alter the proliferation and survival of Kasumi-1 AML cells; a cell line derived from a 7-year-old boy in relapse and used here as a model of t(8;21)-translocated pediatric AML. When compared to a non-targeting scrambled siRNA (SCR), electroporation of Kasumi-1 cells with siRNAs targeting ADARB2-AS1 or LINC01257 led to a significant reduction in their expression within 24 h (Figure 2A). While ADARB2-AS1 knockdown resulted in no change in proliferation of Kasumi-1 cells, LINC01257 knockdown led to a significant progressive decrease in cell proliferation, reaching a 62% and 57% reduction in the total cell count after 48 and 72 h, respectively (Figure 2B). To determine if the observed reduction in proliferation was a result of apoptosis, we performed Annexin V/7AAD- staining on Kasumi-1 cells 48 h following lncRNA knockdown. Unsurprisingly, there was no marked difference in cells entering apoptosis and cell death following ADARB2-AS1 knockdown; however, in the case of LINC01257, the observed suppression of leukemic cell growth was associated with a 42% decrease in cell viability (Figure 2C). These data demonstrate the importance of LINC01257 expression in AML cell survival and prompted us to further investigate the potential of LINC01257 as a therapeutic target.

### 3.3. LINC01257 Is Specifically Expressed in t(8;21) AML and Absent in Healthy Cells

**Figure 3:**
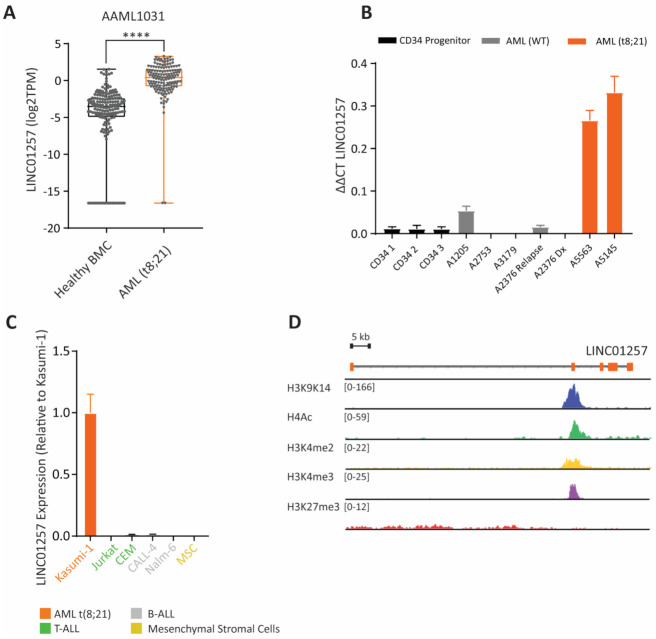
LINC01257 is an AML (t8;21)-specific lncRNA and absent in healthy cells. (**A**) Expression of LINC01257 in healthy bone marrow cells (BMC) and t(8;21) carrying patients from the AAML1031 dataset **** = *p* < 0.0001. (**B**) RT-qPCR analysis of LINC01257 expression in healthy CD34+ cells and AML patient samples with no fusion (WT) and t(8;21). *n* = 3. (**C**) RT-qPCR analysis of LINC01257 expression across a panel of cancer cell lines. t(8;21) positive cell line highlighted in orange. *n* = 3. (**D**) ChIP-seq analysis of histone modification marks in Kasumi-1 cells at the LINC01257 locus.

A major limitation in the discovery of new treatments for AML is finding targets that are specific for AML cells and absent in healthy cells. To further assess the potential of LINC01257 as an AML-specific therapeutic target, we explored whether LINC01257 is specifically found in t(8;21)-translocated AML patients and whether it is differentially expressed in these cells when compared to healthy hematopoietic cells. Firstly, we investigated the expression of LINC01257 in WT vs t(8;21) AML patients from both TARGET and AAML1031 datasets and found that LINC01257 expression was consistently higher in t(8;21) patients across both independent datasets (Supplementary Figure S1A). We also found that the expression of LINC01257 was significantly higher in t(8;21) AML patient bone marrow cells compared to non-leukaemic bone marrow of healthy individuals (Figure 3A). We then aimed to validate our in silico findings by using qRT-PCR to measure LINC01257 expression in pediatric AML mononuclear cell isolates provided by the Children’s Cancer Institute Tumour Bank. Again, LINC01257 expression was found to be specific for t(8;21) AML isolates with 8.7-fold higher expression when compared to WT AML cells. Strikingly, healthy CD34^+^ progenitor cells that give rise to the lymphoid and myeloid lineages expressed negligible levels of LINC01257 (Figure 3B). Similarly, while LINC01257 was highly expressed in Kasumi-1; a t(8;21) AML cell line model, its expression was negligible or absent in a series of non-AML cancer subtypes that included cell lines of acute lymphoblastic leukemia and healthy mesenchymal stroma (MSC) (Figure 3C). Likewise, we measured LINC01257 in a panel of AML cell lines encompassing a diverse range of AML subtypes and demonstrated that Kasumi-1 cells were the only AML cell line that express LINC01257, further highlighting the context-specific expression of lncRNAs in t(8;21) AML (Supplementary Figure S1B). Furthermore, analysis of publicly available ChIP-seq data in Kasumi-1 cells showed a lack of peaks for H3K27me3 (repression mark) and strong peaks for H3K9/K14 (active transcription), H4Ac, H3K4me2 and H3K4me3 (promotor marks) at the LINC01257 locus (Figure 3D) and these were absent in non-AML cell lines (Supplementary Figure S1C). Given LINC01257’s correlation with poor survival, its highly specific expression in t(8;21) AML cells, its absence in stromal and bone marrow progenitor cells, as well as its effect on leukemia cell survival, we decided to develop a new therapeutic model to treat pediatric t(8;21) AML through silencing LINC01257.

### 3.4. Anti-LINC01257 siRNA-Loaded LNPs Are Efficiently Taken Up by Kasumi-1 Cells In Vitro

**Figure 4:**
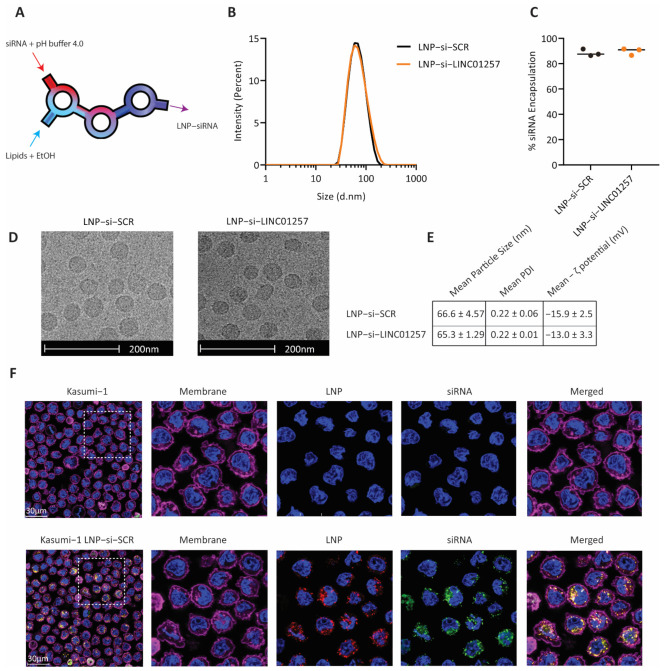
The NxGen non-turbulent microfluidics system generates stable LNP-siRNA particles. (**A**) Schematic of the NxGen non-turbulent microfluidics system. (**B**) Size analysis of LNP-si-SCR compared with LNP-si-LINC01257. (**C**) Average percentage of siRNA encapsulated within LNP of LNP-si-SCR compared with LNP-si-LNC01257 as determined by Ribogreen assay. (**D**) Cryogenic electron microscopy images of LNP-si-SCR and LNP-si-LINC01257. Scale bar: 200 nm. (**E**) Mean particle size, PDI and ζ potential for LNP-si-SCR and LNP-si-LINC01257 (mean ± SEM). *n* = 6. (**F**) Confocal microscopy images of untreated Kasumi-1 cells compared to Kasumi-1 treated with Rhodamine-tagged LNP (red) containing FAM-tagged siRNA (green) for 3 h. Hoechst-stained nuclei (blue). CellMask^®^ Deep Red stained membrane (purple). Scale bar: 30 µM. White box denotes enlarged sections.

As lncRNAs have no protein product they are often considered ‘undruggable’ targets and, consequently, RNAi remains the gold standard method for their targeting. Therefore, we sought to develop an efficient and biologically safe siRNA delivery system using LNPs to target LINC01257 in AML. LNPs were synthesized through NxGen non-turbulent microfluidics to allow a high batch-to-batch reproducibility (Figure 4A). To ensure clinical safety, LNPs were formulated reproducing the FDA-approved Patisiran siRNA delivery vector, i.e., D-Lin-MC3-DMA:DSPC:cholesterol:PEG-DMG (50:10:38.5:1.5) [12]. Rhodamine-labelled lipid-DOPE-Rho was additionally added to the lipid mixture (0.5% total lipid) for LNP imaging. Non-targeting SCR or si-LINC01257 siRNAs were incorporated during LNP manufacturing at a 12:1 lipid:siRNA wt/wt ratio; LNP-siRNA products are henceforth referred to as LNP-si-SCR and LNP-si-LINC01257. A mean LNP size of ~65 nm with PDI ≤ 0.22 (Figure 4B), along with a >85% siRNA encapsulation yield (Figure 4C), were obtained for both LNP-si-SCR and LNP-si-LINC01257, resulting in particles with 0.37 nmol siRNA/µmol lipid. Cryo-Electron Microscopy imaging revealed LNPs of consistent size (~65 nm diameter) and morphology, further supporting the high reproducibility of siRNA-LNP manufacturing using NxGen non-turbulent microfluidics technology (Figure 4D). Of note, LNP-siRNA physicochemical properties were maintained among each batch produced (Figure 4E), which further highlights the reproducibility of NxGen microfluidics. 

A major limitation in delivering siRNA to AML cells is the difficulty of transfecting AML cells using traditional methods. Therefore, we set out to investigate whether siRNA-loaded LNPs associate to and are efficiently taken up by AML cells. To do so, we incubated Kasumi-1 cells for 3 h with LNPs packaging FAM-labelled si-SCR. Flow cytometry analysis revealed that >95% of cells incorporated both the LNP vector and packaged FAM-si-SCR (Supplementary Figure S2A). The intracellular uptake of both the LNP vehicle and FAM-si-SCR cargo was further confirmed by confocal microscopy. Both FAM-si-SCR and the LNP vector were found uniformly distributed in the cell cytoplasm with no observable accumulation in the nucleus (Figure 4F). Furthermore, cell fractionation experiments showed that LINC01257 is abundant in the cell cytoplasmic fraction (Supplementary Figure S2B), and thus is amenable to be targeted via the internalized siRNA. 

### 3.5. si-LINC01257 siRNA-Loaded LNPs Efficiently Ablate LINC01257 Expression

**Figure 5:**
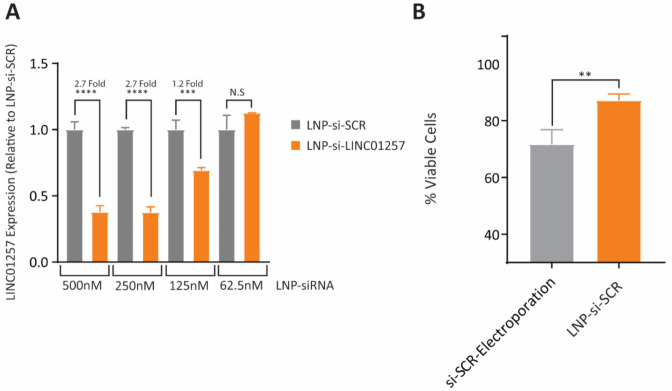
LNP-siRNAs can effectively target LINC01257 in Kasumi-1 cells. (**A**) RT-qPCR of LINC01257 expression following 72 h treatment with LNP-si-SCR at varying concentrations. **** = *p* < 0.0001, *** = *p* < 0.001, N.S = no significance. *n* = 3. (**B**) Viability of Kasumi-1 cells following either electroporation with si-SCR or 72 h LNP-si-SCR treatment. ** = *p* < 0.01. *n* = 3.

To assess whether LNP-mediated si-LINC01257 delivery efficiently inhibits LINC01257 expression in AML cells, we treated Kasumi-1 cells with increasing siRNA concentrations. The largest LINC01257 knockdown was obtained at 500 nM and 250 nM siRNA after 72 h exposure, as determined by RT-qPCR analysis, and this resulted in a significant 2.7-fold reduction in LINC01257 expression when compared to the LNP-si-SCR control (Figure 5A). As no appreciable differences were seen between 500 and 250 nM si-LINC01257, the latter was selected for subsequent experiments. Interestingly, we found LNPs to constitute a less toxic siRNA delivery system than electroporation, which is the most commonly utilized approach for siRNA transfection in AML [26]. SCR siRNA delivery through LNPs resulted in a minimal 10% decrease in Kasumi-1 viability, compared to a total 30% decrease when using electroporation (Figure 5B and Figure S2C). Together, these data confirm that LNPs are an efficient and low-toxic approach to deliver siRNA into human AML cells and can efficiently ablate lncRNA expression in these cells. 

### 3.6. LNP-si-LINC01257 Impairs Kasumi-1 Cell Proliferation without Affecting Healthy PBMCs

**Figure 6:**
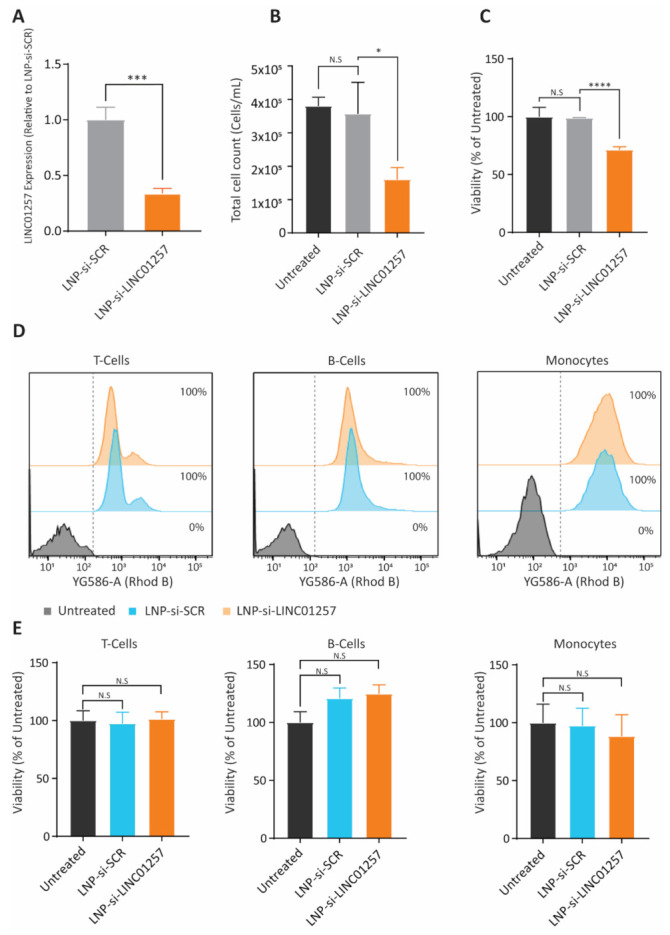
LNP-si-LINC01257 inhibits growth of Kasumi-1 cells but not healthy PBMCs. (**A**) RT-qPCR analysis of LINC01257 expression in Kasumi-1 cells treated with LNP-si-SCR or LNP-si-LINC01257. *** = *p* < 0.001. (**B**) Cell counts of Kasumi-1 cells 48 h following LNP-si-SCR or LNP-si-LINC01257 treatment. * = *p* < 0.05, N.S = no significance. (**C**) Percentage of viable Kasumi-1 cells following treatment with LNP-si-SCR or LNP-si-LINC01257. Untreated cells were used as a normalized control. **** = *p* < 0.0001, N.S = no significance. (**D**) Flow cytometry histograms of LNP-si-SCR and LNP-si-LINC01257 uptake, indicated by Rhodamine B (Rhod B) signal, in T-cell, B-cell and monocyte populations from healthy donor PBMCs following 48 h treatment. (**E**) Viability of T-cell, B-cell and Monocytes following 48 h treatment with LNP-si-SCR or LNP-si-LINC01257. Untreated cells were used as a normalized control. N.S = no significance. *n* = 3.

Following confirmation that treatment with LNP-si-LINC01257 can reduce LINC01257 expression in Kasumi-1 AML cells, we next investigated the effects of LNP-si-LINC01257 on Kasumi-1 cell growth. LNP-si-LINC01257 treatment resulted in a 2.9-fold reduction in LINC01257 expression (Figure 6A), a 55% reduction in proliferation of Kasumi-1 (Figure 6B) along with a 29% decrease in cell viability (Figure 6C) as a consequence of treatment inducing apoptosis and cell death (Supplementary Figure S3A). These results were comparable to those observed using the traditional siRNA delivery system of electroporation. Of note, Kasumi-1 exposure to LNP-si-SCR control caused no differences in leukemia cell growth or viability as compared to untreated cultures, which suggests that LNP-si-LINC01257 causes a cytotoxic response on these cells through targeted silencing of LINC01257 (Figure 6B,C).

A major cause of morbidity and mortality of conventional AML therapies is their toxic immunosuppressive action due to off-target effects on healthy blood mononuclear immune cells [2]. Therefore, we sought to determine whether LNP-si-LINC01257 causes a decrease in viability of PBMCs isolated from healthy donors. Healthy PBMCs were treated with either LNP-si-LINC01257 or LNP-si-SCR control and the T cell, B cell and monocyte populations were analyzed for LNP-siRNA uptake. The effects of treatment with LNP-si-LINC01257 vs. LNP-si-SCR on cell viability were further analyzed 48 h after (Supplementary Figure S3B). We found a significant association of both LNP-si-SCR and LNP-si-LINC01257 with PBMC populations, with >99% of B cells, T cells and monocytes being targeted by LNP-si-SCR and LNP-si-LINC01257 (Figure 6D and Figure S4A). However, in stark contrast to Kasumi-1 cells, where LNP-si-LINC01257 significantly decreased cell growth and viability, treatment with LNP-si-LINC01257 exerted a negligible impact on the viability of all three PBMC populations, with no difference in viability observed in monocytes and T cells, and a small but non-significant increase in B cell viability (Figure 6E and Figure S4B). These promising results provide preliminary evidence for the safety of LINC01257 silencing through LNP-assisted RNAi and provide insight into the therapeutic advantage of targeting lncRNAs related to chromosomal translocations as a precision medicine approach for children with AML.

## 4. Discussion

Conventional chemotherapies in AML target healthy mononuclear cells and there are only a small number of AML-specific treatments that have been approved for clinical use [27]. The clinical effectivity of other emerging technologies, such as CAR-T cell therapy, is also limited due to their interference with normal hematopoiesis [3]. The immunosuppressive effects of these available treatments are linked to higher rates of infections, being a major cause of death in children with AML. Consequently, there is a need to identify new therapeutic strategies for pediatric AML that are both safe and specific to cancer cells. 

Due to their biological significance and exclusive expression in leukemia, chromosomal translocations are attractive therapeutic targets. However, there are key limitations in their direct targeting by conventional drug discovery approaches. Namely, the exact location of gene fusion can differ greatly between patients, consequently limiting the effectiveness of targeted therapies to a patient-specific context [6]. Furthermore, AML fusions consist of genes that have functional wild type versions in healthy tissue; thus, any therapy targeting the fusion is likely to have off-target effects. Considering these points, novel AML therapies might be better suited to target key downstream elements activated by oncogenic fusion proteins.

The expression of oncogenic lncRNAs is in many cases associated with the presence of chromosomal translocations that are specific to cancer cells, which, along with their absence in healthy tissues, makes these ‘onco-lncRNAs’ attractive therapeutic targets with a potentially lower risk of toxic side effects [28]. Onco-lncRNAs have been investigated in other cancers such as breast and lung cancer, demonstrating the potential of these molecules as therapeutic targets [29,30]. Likewise, the expression of specific lncRNA subsets has been identified as a result of chromosomal translocations that are uniquely found in patients with AML [8]. Here, we identified a novel lncRNA, LINC01257, whose expression is associated with the t(8;21) chromosomal translocation, which is specific to AML and occurs with a high incidence in children with AML [22]. This finding is consistent with previous studies which report the subtype-specific nature of lncRNA expression in AML [31,32,33]. Furthermore, LINC01257 expression was absent in healthy progenitor CD34^+^ stem cells, MSCs and other subtypes of leukemia, highlighting the specificity of LINC01257 for AML and the value of its targeting as a precision medicine approach for this disease. LINC01257 expression is also associated with reduced event-free survival in children, demonstrating that LINC01257, and possibly other lncRNAs, can be utilized as both therapeutic targets and prognostic biomarkers in pediatric AML. 

While the majority of lncRNAs in the human genome have no known function, there has been a small number of lncRNAs with identified roles in cancer [34,35]. Interestingly, the vast majority of lncRNAs identified from the bioinformatic analysis performed in this study have no reported function. Moreover, LINC01257, the key lncRNA of this study, has no previously described function in any tissue, much less AML. While further studies are needed to determine the precise mechanism by which LINC01257 regulates AML cell growth and survival, as well as the role of LINC01257 in other tissue types, this study highlights the limited knowledge we currently possess regarding the role and function of lncRNAs in pediatric AML and demonstrates the importance of further research into the therapeutic potential of lncRNAs in pediatric AML.

RNAi has the capacity to theoretically target any oncogenic lncRNA present in AML [36]. Indeed, RNAi has the potential to surpass the major limitations of conventional leukemia therapies including severe off-target toxicity to healthy tissues and peripheral immune cells, a low specificity for cancer cells, and the inability to inhibit non-druggable targets, such as lncRNAs. However, there are many challenges in the development of siRNA-based medicines. For example, a major handicap in RNAi therapy is the intracellular delivery of siRNA. Factors such as enzymatic degradation and entrapment by phagocytes stand as major roadblocks to effective siRNA action [37]. Furthermore, upon reaching the cancer cell, siRNAs cannot efficiently internalize due to the charge barrier posed by the negatively charged cellular membrane. LNPs represent an excellent system for siRNA delivery that has been successfully utilized in a range of adult cancers [38], yet their efficacy in AML remains poorly studied. Another major challenge in the development of siRNA-based medicine is ensuring that the siRNA and LNP vehicle are safe for use and have reduced off-target toxicity. In order to overcome these challenges, we developed LNPs, following the clinically safe lipid formulation of LNP-siRNA Patisiran, as a delivery vehicle of choice for anti-LINC01257 siRNA. This system has received FDA-approval for the treatment of polyneuropathy caused by hereditary amyloidosis [12,13]. Pioneering studies have provided preliminary evidence for the therapeutic efficacy of Patisiran-based LNP formulations to silence oncogenes in lymphoma and chronic myeloid leukemia [18,39,40]. Based on its clinical significance, Patisiran-based LNP formulations set out a standard model of use for other hematological malignancies, such as AML, and will considerably advance the clinical translation of novel siRNA targeted therapies in the management of pediatric cancers [13].

An additional challenge in translating LNP-assisted RNAi therapy into a clinical settings is the ability to produce particles of consistent size and ensure high siRNA encapsulation efficiency in a scalable fashion so as to ensure therapeutic performance is maintained [41]. This limitation has been overcome thanks to the advancement of microfluidic mixing technologies based on staggered herringbone micromixers [42]. The NanoAssemblr NxGen toroidal microfluidic mixer provides further advancements in scaling-up up LNP-siRNA manufacturing by enabling the processing of larger volumes using higher flow rates (>20 mL/min) within a single micromixer [43]. In this study, we made use of NxGen microfluidic technology to precisely generate LNPs with high batch-to-batch reproducibility and ensure the correct manufacturing of larger volumes needed to progress into in vivo assays [18]. This technology also has the potential for use in the delivery of other nucleic acids such as plasmid DNA and antisense oligonucleotides (ASOs) [44], thereby broadening the applications of LNP-based therapies in AML. Finally, the accumulation of LNP-siRNA particles in the liver following a systemic delivery presents a major challenge with siRNA-based therapies. While this provides excellent efficiency of cargo delivery to hepatic diseases, it limits the application of LNP-siRNA based therapies in other tissues [45]. To overcome this limitation, targeting ligands can be implemented into the formulation of the LNP to enhance delivery to other organs; however, this adds additional regulatory barriers and increases the costs and the difficulty of LNP development [46]. 

This study is the first to present the use of LNP-assisted RNAi in the management of pediatric AML. Our synthesized LNPs were able to achieve near 100% uptake and siRNA delivery in difficult-to-transfect AML cells [47] and demonstrated LINC01257 knockdown efficiency comparable to conventional methods. However, LNP-assisted siRNA delivery was found to be safer than conventional electroporation methods and caused negligible cytotoxicity alone, highlighting the value of LNPs and the combined use of D-Lin-MC3-DMA/PEG-DMG as cationic/stealth phospholipids, as a safe and robust siRNA delivery system. LINC01257 silencing through LNP-si-LINC01257 effectively reduced AML cell proliferation and viability while having no notable toxicity on healthy blood mononuclear cells. These findings demonstrate the value of targeting cancer-specific lncRNAs through LNP-assisted RNAi as a feasible therapeutic strategy and provide a glimpse into the potential absence of immunosuppressive effects within a clinical setting. Interestingly, the high uptake efficiency of LNPs observed in T-cell, B-cell and monocyte populations emphasizes the usefulness of LNPs in not only targeting AML cells but also delivering siRNAs to other hematopoietic populations.

While we observed a promising therapeutic efficacy of LNP-si-LINC01257 towards t(8;21) AML cells and an absence of toxicity on healthy human cells in vitro, a limitation of this study is the reliance of the Kasumi-1 AML cell line as our primary model. Cell lines can have biological differences compared to the original cancer cells due to the artificial nature of their culture conditions. Therefore, future studies in animal models of the disease are needed to delineate the therapeutic benefit of LNP-si-LINC01257 within an in vivo setting and to further assess any potential off-target effects on healthy tissues in a more translational setting. Furthermore, Onpattro-like siRNA-LNP formulations are rapidly taken up by the liver [45,48], thereby reducing the amounts of siRNA-LNPs in circulation available to target other sites in the organism. Prior to a full assessment of our strategy in vivo, improvements on LNP selectivity toward AML cells by introducing AML-specific targeting ligands, and establishing dosages with best circulation times, will be necessary to ensure the delivery of lethal doses of anti-LINC01257 to the circulating AML cells. 

Finally, the success observed with LNP-si-LINC01257 in AML suggests that analogous strategies targeting cancer-specific lncRNAs can be further expanded to treat other leukemia subtypes and diseases driven by lncRNAs. However, this study provides a robust proof of principle model for the therapeutic benefits and safety of lncRNA silencing through LNP-assisted siRNA delivery.

## 5. Conclusions

In this study, we analyzed the expression of lncRNAs in pediatric AML patients and identified LINC01257 as an lncRNA that plays a key role in the survival and proliferation of pediatric t(8;21) AML. Using LNP-siRNAs synthesized by NanoAssemblr NxGen toroidal microfluidic mixer technology, we demonstrated the efficient uptake of LNP-si-LINC01257 into a cell line model of pediatric AML and showed this was coupled with the ablation of LINC01257 expression. Importantly, LNP-si-LINC01257 had no effect on healthy PBMCs which do not express LINC01257. In conclusion, this study validates the therapeutic benefits and safety of lncRNA silencing through LNP-assisted siRNA delivery. 

## Data Availability

The data presented in this study are available on request from the corresponding author.

## Supplementary Materials

The following are available online at https://www.mdpi.com/article/10.3390/pharmaceutics13101681/s1, Figure S1: LINC01257 is an AML t(8;21)-specific lncRNA, Figure S2: LNP-siRNA are non-toxic and efficiently taken up by Kasumi-1 cells, Figure S3: Flow cytometry gating of Kasumi-1 and PBMCs, Figure S4: PBMCs efficiently take up and are unaffected by LNP-siRNAs.
