# Supplementary material for: Development of siRNA-Loaded Lipid Nanoparticles Targeting Long Non-Coding RNA LINC01257 as a Novel and Safe Therapeutic Approach for t(8;21) Pediatric Acute Myeloid Leukemia
Source: Pharmaceutics. 2021 Oct 14;13(10):1681. doi: 10.3390/pharmaceutics13101681 (PMC8539450; doi:10.3390/pharmaceutics13101681)
Supplement: Supplementary file 1 [file pharmaceutics-13-01681-s001.zip › pharmaceutics-1363985-supplementary.pdf]

pharmaceutics

Supplementary Materials: Development of siRNA-Loaded Lipid Nanoparticles Targeting Long Non-Coding RNA LINC01257 as a Novel and Safe Therapeutic Approach for t(8;21) Pediatric Acute Myeloid Leukemia

Patrick Connerty, Ernest Moles, Charles E. de Bock, Nisitha Jayatilleke, Jenny L. Smith, Soheil Meshinchi, Chelsea Mayoh, Maria Kavallaris and Richard B. Lock *

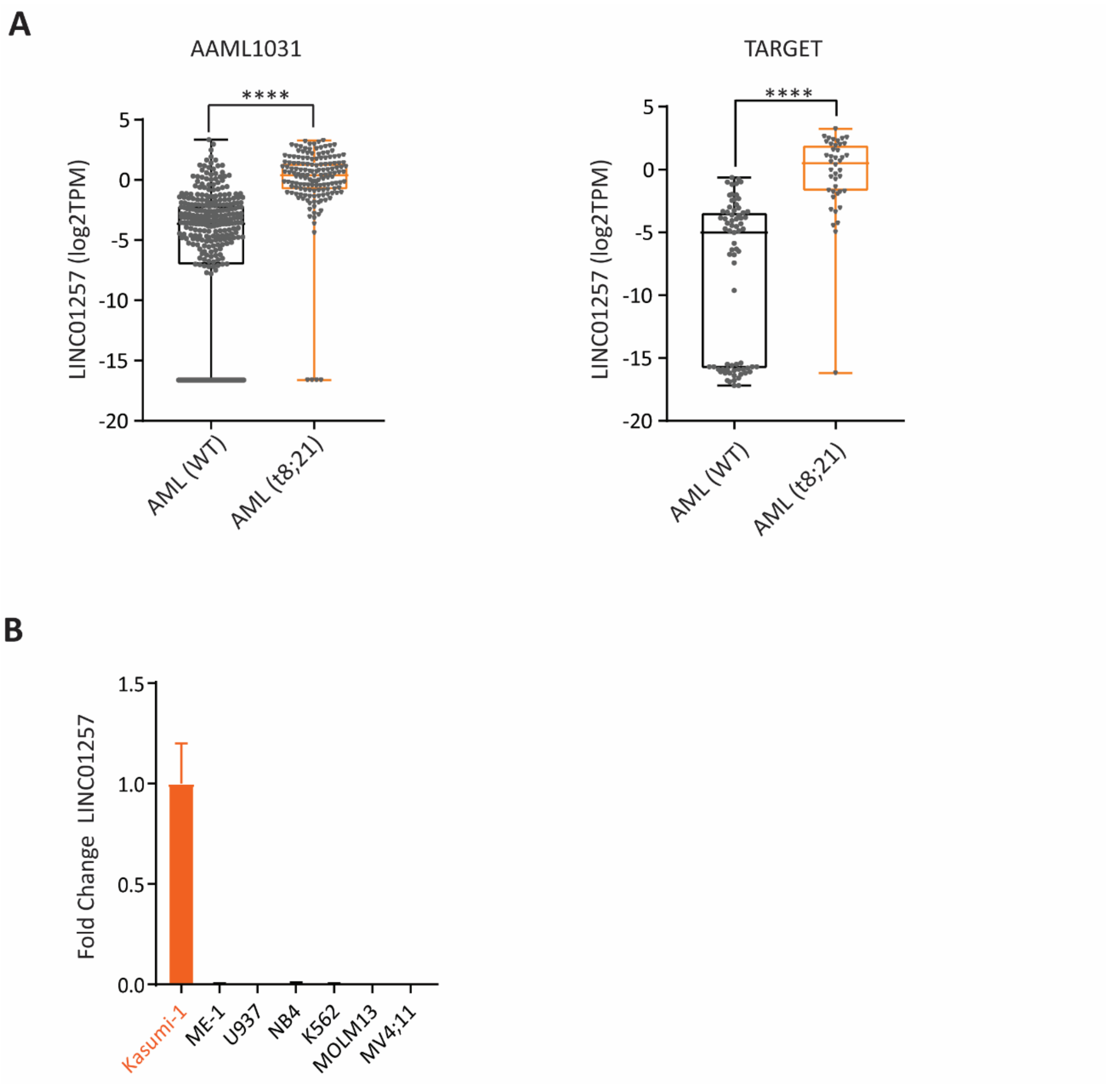

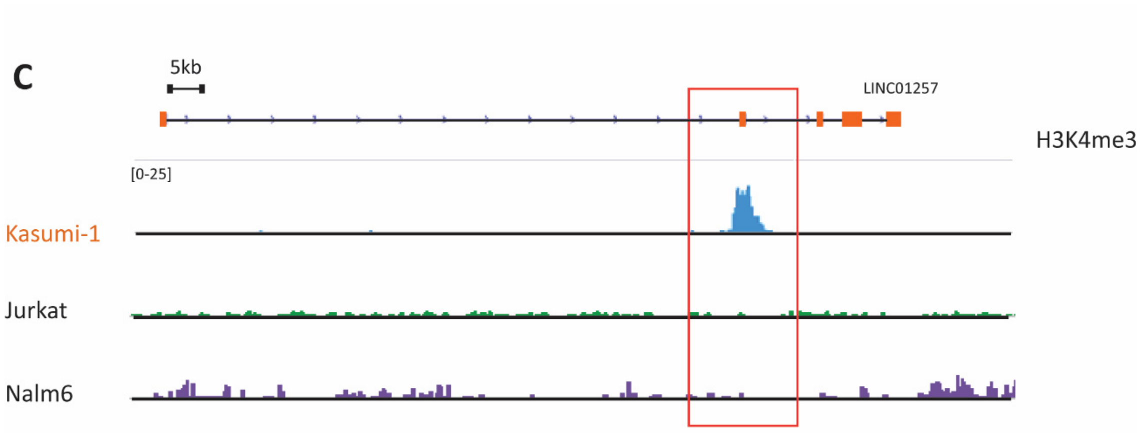

Figure S1. LINC01257 is an AML t(8;21) specific lncRNA. (A) Expression of LINC01257 in fusionless wild type (WT) karyotype and t(8;21) carrying patients from AAML1031 and TARGET datasets **** = $p < 0.0001$. **(B)** Expression of LINC01257 in panel of AML cell lines. t(8;21) AML cell line denoted in orange. **(C)** ChIP-seq analysis of H3K4me3 methylation across Kasumi-1 (AML), Jurkat (T-ALL) and Nalm6 (B-ALL) cell lines. Peak highlighted by orange box. t(8;21) AML cell line denoted in orange.

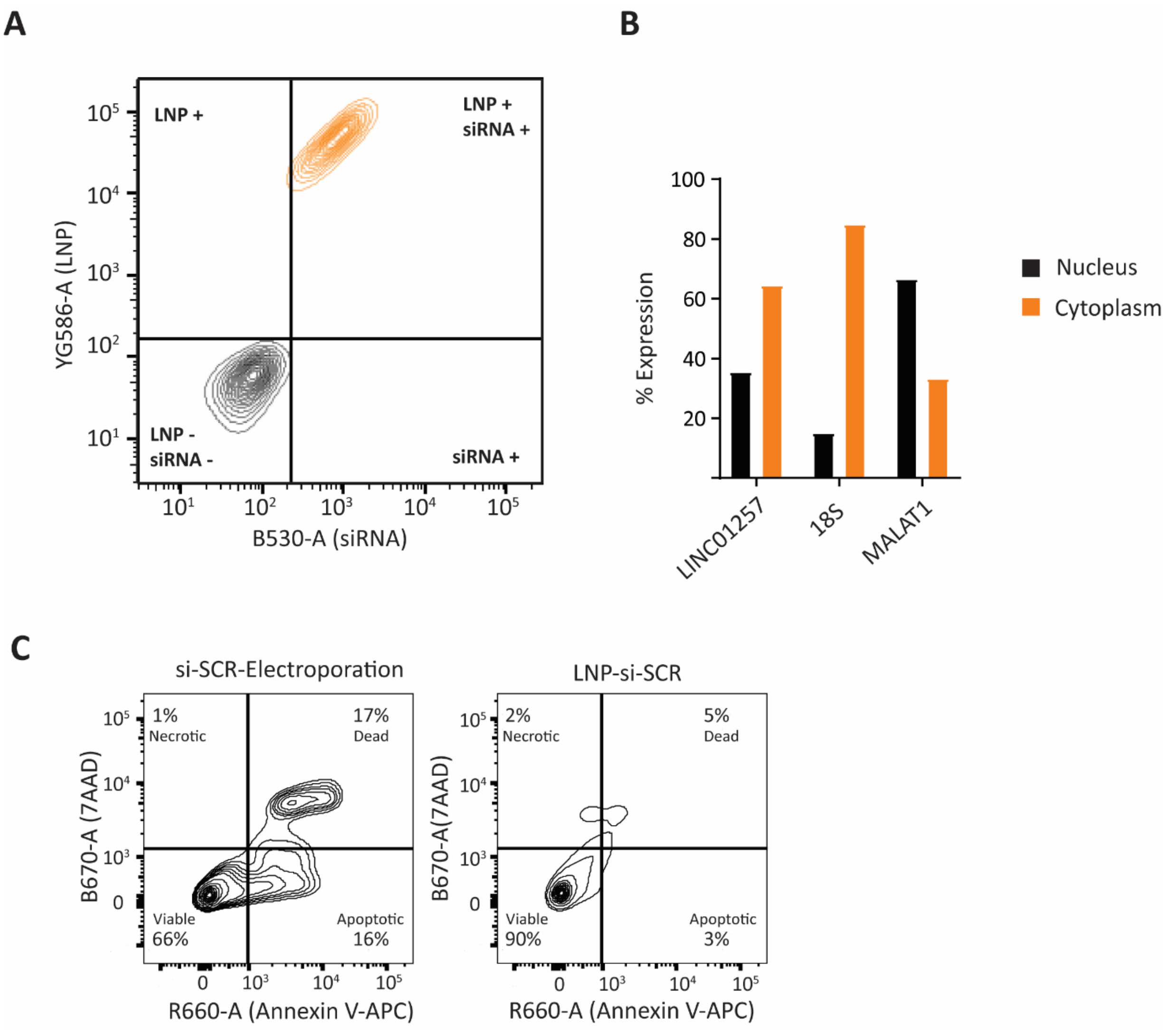

Figure S2. LNP-siRNA are non-toxic and efficiently taken up by Kasumi-1 cells. (A) Representative flow cytometry analysis of Kasumi-1 cell uptake of LNP-siRNAs. **(B)** RT-qPCR data from cytoplasmic and nuclear fractions of Kasumi-1 cells normalized as a total percentage. 18s and MALAT1 were used as cytoplasmic and nuclear controls, respectively. **(C)** Representative flow cytometry plots of Kasumi-1 cells stained with Annexin V/7AAD following electroporation or 72 h LNP-si-SCR treatment.

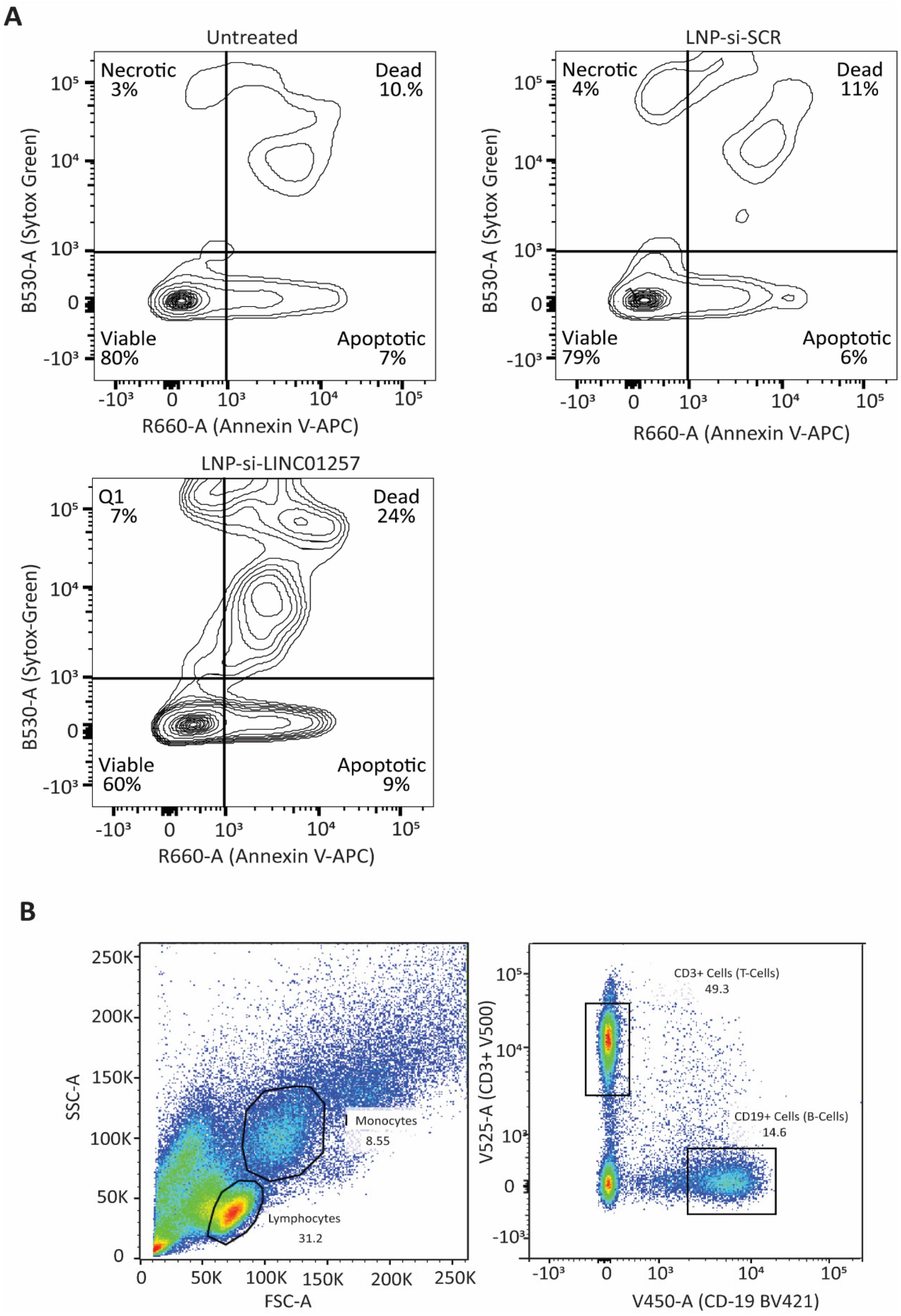

Figure S3. Flow cytometry gating of Kasumi-1 and PBMCs. (A) Representative flow cytometry plots of Kasumi-1 cells stained with Annexin V/Sytox following 48 h LNP-siRNA treatment vs untreated controls. **(B)** Representative flow plots of healthy PBMCs demonstrating monocyte, T-cell and B-cell populations.

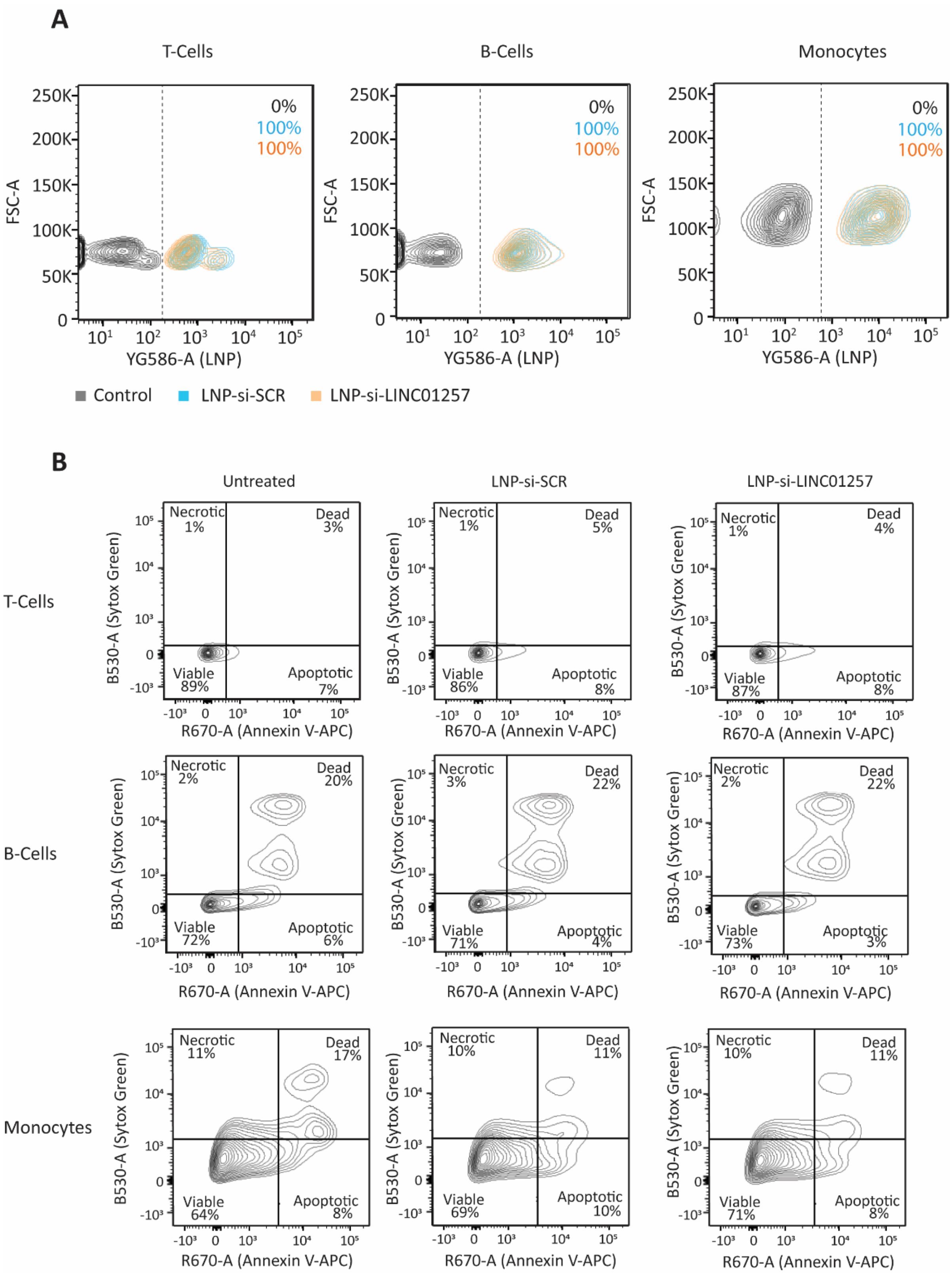

Figure S4. PBMCs efficiently take up and are unaffected by LNP-siRNAs. (A) Representative flow cytometry plots of LNP-si-SCR and LNP-si-LINC01257 uptake in T-cell, B-cell and monocyte populations from healthy donor PBMCs. **(B)** Representative flow cytometry plots showing the viability of T-cell, B-cell and monocytes treated with LNP-si-SCR or LNP-si-LINC01257 for 48 h compared to untreated controls.
